# Moving Beyond Directly Observed Therapy for Tuberculosis

**DOI:** 10.1371/journal.pmed.1001877

**Published:** 2015-09-15

**Authors:** John Z. Metcalfe, Max R. O’Donnell, David R. Bangsberg

**Affiliations:** 1 Division of Pulmonary and Critical Care Medicine, San Francisco General Hospital, University of California, San Francisco, San Francisco, California, United States of America; 2 Division of Pulmonary, Allergy, and Critical Care Medicine, Columbia University Medical Center, New York, New York, United States of America; 3 Massachusetts General Hospital, Harvard Medical School, Harvard School of Public Health, Boston, Massachusetts, United States of America; 4 Mbarara University of Science and Technology, Mbarara, Uganda

*Mycobacterium tuberculosis* often develops resistance in the setting of monotherapy, either de facto (when an organism is susceptible to only one drug of an intended multidrug regimen) or actual (historically, or in the setting of extensively drug resistant tuberculosis (XDR-TB) salvage regimens) [1–4]. With current regimens, sterilization of drug-susceptible organisms requires at least six months of treatment to prevent disease relapse. Unfortunately, pill burden, drug toxicity, stigma, and poor provider practice often complicate these prolonged treatment courses, and nonadherence is widely blamed for the global epidemic of drug-resistant TB. Directly observed therapy (DOT) has for decades been considered crucial to ensuring anti-TB medication adherence worldwide, but carries important individual and health policy concerns. This week in *PLOS Medicine*, Fielding and colleagues present a cluster randomized trial of an alternative strategy—managing adherence with reminders delivered by an electronic pillbox or text messaging.

DOT is variously defined as 5–7 daily doses per week observed by facility-, workplace-, or community-based healthcare workers or confidants. Although DOT is employed selectively for other communicable and noncommunicable diseases for which incomplete adherence threatens treatment success, DOT in TB treatment has become enshrined as “canon in a field long characterized by fervor in principle and practice” as in no other disease [5]. First implemented in Madras and Hong Kong in the 1950s [6], DOT became globally endorsed (as one of five key components of the World Health Organization directly observed therapy short-course [DOTS] strategy) [7] in 1994 in the aftermath of public health alarm created by an outbreak of multidrug-resistant (MDR)-TB in New York City [8]. Operationalization of DOT varies according to resource availability, cultural factors, and individual provider perception, and is often incomplete, even among populations for which strict adherence is considered essential [9]. Although in its best form DOT can be a platform for patient social support and guidance, it has long been debated whether the requirement for witnessed dosing represents the least restrictive alternative in pursuit of public health goals [10]. Conceived of as a principal standard of care to protect individuals from drug resistance amplification and communities from a potentially devastating airborne disease, DOT in some cases adversely impacts the dignity, autonomy, and livelihoods of patients who are often already poor and disenfranchised [11]. For example, among patients receiving treatment for both HIV and drug-resistant TB, loss of a sense of agency due to provider supervision of TB treatment contributes to preferential adherence to antiretrovirals over drug-resistant TB therapy, complicating combined treatment regimens that may surpass 30 pills daily [12]. Nevertheless, alternative strategies to DOT have received little attention.

To what degree does nonadherence lead to treatment failure or acquisition of drug resistance? In contrast to HIV, for which the complex relationship between adherence, pharmacokinetics, and resistance for each antiretroviral class is defined [13], the levels and patterns of adherence that lead to TB treatment failure and drug resistance remain largely unknown [14]. Observational studies from the United States [15] and Botswana [16] in the 1990s documented associations between DOT-supported adherence and prevention of drug resistance. However, a meta-analysis of randomized trials [17] comparing DOT to self-administration of TB treatment failed to demonstrate improved treatment outcome (though the component trials were heterogeneous [18] and were not powered to examine drug resistance amplification). In addition, preclinical studies suggest that TB treatment is robust to relatively high levels of nonadherence [19], and certain drugs and drug combinations are more or less forgiving than others [4,20]. Nevertheless, adherence challenges with prior TB treatment are a common refrain among people living with MDR-TB [21].

The study by Fielding and colleagues was a four-arm cluster randomized trial, involving 4,173 patients in 36 health centers across China, designed to assess the effect of dosing and refill reminders delivered by cell phone text message, electronic pillbox, or both, on patient adherence to six months of intermittent (every other day) TB treatment. The primary outcome was the proportion of patient-months in which ≥20% of doses were missed (“nonadherent months”), measured by either monthly pill counts or electronic pillbox openings (which did not produce audible reminders in the control arm of the study). Despite use of medication pillboxes, various forms of DOT, and data-informed (pill counts) counseling in the control arm, substantial nonadherence was observed (30% nonadherent months). Electronic pillbox reminders, but not text messages alone, significantly decreased the primary outcome (17% nonadherent months). This effect was stronger when text message reminders were added to pillbox reminders (14% nonadherent months). Approximately one-tenth of patients were excluded because of inability to use mobile phones after training, and nearly one-third of randomized patients (and one-half of those in the combined text messaging and electronic pill box arm) experienced technical problems with their device (S5 Table in the supplementary material of the paper by Fielding and colleagues). Despite substantial nonadherence to an intermittent regimen, adverse end-of-treatment outcomes, other than loss to follow up, were rare in this low HIV burden setting.

Significant technical complications in the trial related to battery connectivity suggest that additional work is needed to both confirm benefit and facilitate scale-up, and the data provide little indication of whether durable effects on treatment outcomes (beyond loss to follow-up) should be anticipated. Yet, the study by Fielding and colleagues is unusual in providing objective TB treatment adherence data combined with a scalable intervention strategy to improve adherence. If replicated, it will have important implications for global TB treatment in moving away from witnessed dosing, which is not universally feasible, towards a more personalized adherence model of patient–provider communication in which intervention is delivered where, when, and in whom it is needed to efficiently prevent adverse treatment outcome.

Will such technology aid transition to a “post-DOT” era in low- as well as high-income settings? In the field of HIV, “just-in-time” adherence interventions linked in real-time to objectively monitored adherence have overcome several limitations in traditional counseling-based interventions [22], which cannot anticipate decline in adherence over time or interruptions in daily adherence routines. Such approaches can potentially reduce healthcare costs by selectively targeting other medical resources (e.g., laboratory monitoring, provider visits, pharmacokinetic testing) to patients at greatest risk, while foregoing these for patients with near-perfect adherence and negligible risk of disengagement, treatment failure, or drug resistance [23].

In its long history with humanity, tuberculosis has in many ways provided the prototype for chronic disease management. The cornerstone of successful TB treatment has long been recognized to lie within the complex inter-relationship between patients and clinical staff and to hinge strongly on structural facilitators to the treatment experience, along with empathy [24]. Shortened, simplified TB treatment regimens in conjunction with technologies facilitating adherence, such as real-time adherence monitoring and novel drug-delivery systems, could support this foundation to speed TB elimination while delivering truly patient-centered care.

## Abbreviations

DOT: directly observed therapy
DOTS: directly observed therapy short-course
MDR: multidrug-resistant
TB: tuberculosis
